# Supplementary material for: Evaluation of Physicochemical Characteristics and Sensory Properties of Cold Brew Coffees Prepared Using Ultrahigh Pressure under Different Extraction Conditions
Source: Foods. 2023 Oct 21;12(20):3857. doi: 10.3390/foods12203857 (PMC10606293; doi:10.3390/foods12203857)
Supplement: Supplementary file 1 [file foods-12-03857-s001.zip › foods-2628532-supplementary.pdf]

Table S1. Volatile components of UHP-assisted cold brew coffee under different pressures

Aroma Type	Compound Name	Aroma descriptor	Content/(μg/L)					
			Pressure/(MPa)					Control group
			100	200	300	400	500	
Furans	2-methylfuran	Chocolate	2.59±0.10^{a}	1.19±0.06^{d}	1.13±0.03^{d}	1.98±0.10^{b}	1.39±0.05^{c}	1.56±0.11^{c}
	2, 5-dimethylfuran	Meaty, beef, bacon	0.25±0.01^{d}	0.25±0.01^{d}	0.49±0.03^{c}	0.62±0.02^{b}	0.99±0.07^{a}	0.30±0.02^{d}
	furfural	Almond, bread	75.71±2.73^{d}	77.57±3.08^{d}	80.53±4.20^{c}	82.58±4.35^{bc}	93.07±4.29^{a}	86.46±3.83^{b}
	2-acetyl-5-methylfuran	Nutty, vanilla, coconut	-	0.39±0.01^{c}	0.48±0.04^{b}	0.96±0.07^{a}	0.51±0.02^{b}	0.98±0.05^{a}
	2-propionylfuran	Fruit	-	4.25±0.15^{b}	4.79±0.13^{a}	4.64±0.20^{ab}	3.06±0.11^{c}	5.06±0.22^{a}
	2-(furan-2-methyl-furan)	Roasted	2.27±0.10^{c}	2.29±0.13^{c}	2.58±0.11^{b}	2.46±0.09^{bc}	2.88±0.12^{a}	3.16±0.13^{a}
	Furfuryl alcohol	Caramel	21.90±1.54^{b}	23.31±1.21^{a}	23.51±1.87^{a}	24.21±1.33^{a}	24.55±1.49^{a}	25.33±1.77^{a}
	2-acetylfuran	Sweet, almond, cocoa, caramel	15.88±0.66^{c}	17.60±0.78^{b}	17.44±0.92^{b}	18.59±0.71^{ab}	20.85±0.88^{a}	19.91±0.76^{a}
	Total		118.61±6.72^{d}	126.86±7.05^{c}^{d}	130.93±5.95^{abc}	136.04±5.47^{b}	147.32±6.84^{a}	142.74±8.43^{a}^{b}
Aldehydes	2-methylbutyral	Cocoa, coffee, nutty	6.16±0.23^{c}	6.32±0.35^{c}	7.59±0.42^{b}	8.35±0.52^{ab}	8.18±0.47^{ab}	9.07±0.63^{a}
	isoamylaldehyde	Chocolate	6.40±0.30^{d}	6.73±0.38^{d}	6.81±0.35^{d}	8.89±0.44^{b}	9.75±0.39^{a}	7.80±0.35^{c}
	Hexal (aldehyde C-6)	Green, fruity	0.98±0.03^{a}	0.56±0.02^{c}	1.11±0.04^{a}	0.69±0.01^{b}	0.40±0.02^{c}	0.39±0.01^{c}
	benzaldehyde	Bitter almond odor, nutty	2.67±0.09^{a}	2.04±0.06^{b}	1.95±0.07^{bc}	1.83±0.04^{c}	1.93±0.03^{bc}	2.53±0.06^{a}
	5-methylfurfural	Spicy, caramel, maple	64.96±4.28^{b}	67.29±3.96^{b}	66.64±4.72^{b}	65.19±4.20^{b}	69.88±3.78^{b}	80.26±4.23^{a}
	1-methyl-2-	Roasted, nutty	9.25±0.44^{c}	10.31±0.42^{bc}	10.20±0.55^{bc}	9.32±0.54^{c}	11.33±0.62^{a}	11.85±0.45^{a}

	pyrroformaldehyde							
	Total		90.42±4.92^{c}	93.26±6.43^{bc}	94.30±5.39^{bc}	94.25±4.92^{bc}	101.48±5.34^{ab}	111.89±6.53^{a}
Esters	Glycol diacetate	Green, flowery, alcoholic	2.51±0.03^{a}	1.69±0.02^{b}	1.61±0.02^{b}	1.79±0.03^{b}	1.75±0.02^{b}	2.60±0.04^{a}
	Furfuryl acetate	Sweet, banana	67.06 ±4.29^{b}	69.37 ±3.88^{b}	67.21 ±4.04^{b}	67.63 ±3.90^{b}	71.64 ±3.76^{ab}	78.61 ±4.19^{a}
	Furfuryl propionate	Sweet, fruit, banana	2.70±0.04^{c}	3.14±0.10^{b}	3.11±0.09^{b}	3.04±0.07^{b}	2.34±0.05^{d}	4.35±0.12^{a}
	Total		72.27±4.33^{b}	74.20±4.50^{b}	71.94±5.13^{b}	72.46±4.85^{b}	75.73±5.31^{b}	85.57±6.45^{a}
Pyrazines	2, 5-dimethylpyrazine	Cocoa, nutty, beef	6.27±0.33^{d}	7.17±0.36^{c}	7.82±0.52^{b}	8.10±0.41^{ab}	8.90±0.61^{a}	8.33±0.39^{ab}
	2, 6-dimethylpyrazine	Cocoa, roasted, nutty	9.41±0.67^{a}	7.83±0.40^{b}	7.19±0.33^{c}	7.47±0.43^{bc}	7.86±0.42	7.89±0.53^{b}
	2-ethylpyrazine	Peanut, butter, nutty, cocoa	5.39±0.32^{c}	6.44±0.39^{b}	6.74±0.37^{ab}	6.63±0.43^{ab}	7.41±0.48^{a}	7.36±0.45^{a}
	2, 3-dimethylpyrazine	Nutty, cocoa, peanut	0.56±0.02^{b}	0.58±0.01^{b}	0.99±0.05^{a}	0.88±0.02^{a}	0.61±0.02^{b}	0.61±0.03^{b}
	2-ethyl-6-methylpyrazine	Potato	11.93 ±0.53^{d}	12.49 ±0.61^{cd}	12.96 ±0.67^{c}	11.83 ±0.59^{d}	14.11 ±0.72^{b}	15.88 ±0.65^{a}
	Hazelnut pyrazine	Nutty, peanut, corn, bread	2.40±0.12^{e}	3.14±0.21^{d}	4.97±0.27^{c}	5.73±0.28^{b}	6.70±0.45^{a}	6.09±0.44^{ab}
	3, 6-theopyrazine	Potato, cocoa, nutty	4.80±0.31^{c}	5.84±0.33^{b}	4.96±0.37^{c}	5.98±0.45^{b}	6.61±0.39^{ab}	7.20±0.52^{a}
	Total		40.78±2.49^{c}	43.47±3.01^{bc}	45.63±2.88^{b}	46.63±2.97^{b}	52.22±3.85^{a}	53.34±4.13^{a}
Alcohols	hexanol	Alcoholic	0.77±0.02^{b}	0.89±0.01^{a}	0.85±0.01^{a}	0.90±0.02^{a}	0.91±0.03^{a}	0.69±0.02^{c}
	(E) -linalool oxide (furanoic acid)	Flowery	4.72±0.25^{d}	5.24±0.33^{c}	5.78±0.39^{bc}	5.69±0.31^{bc}	6.38±0.41^{b}	7.75±0.40^{a}
	2-ethyl-1-hexanol	Oranges, flowery, sweet	-	0.60±0.02^{c}	0.59±0.01^{c}	0.71±0.03^{b}	0.80±0.03^{a}	0.59±0.01^{c}
	linalool	Oranges, flowery, sweet, rose	6.70±0.44^{d}	7.45±0.39^{c}	7.79±0.52^{bc}	7.82±0.47^{bc}	8.31±0.55^{b}	12.93±0.73^{a}
	alpha-terpineol	Pine, terpene, lilac, Oranges, flowery	2.05±0.09^{bc}	2.16±0.12^{b}	2.10±0.10^{b}	1.93±0.15^{c}	2.20±0.11^{b}	3.24±0.17^{a}

	nerolol	Sweet, oranges		-	0.39±0.01^{c}	0.38±0.02^{c}	0.48±0.02^{b}	0.69±0.03^{a}
	Total		14.25±0.95^{d}	16.36±0.73^{c}	17.52±0.98^{bc}	17.44±1.05^{bc}	19.10±1.47^{b}	25.89±1.59^{a}
Ketones	Methyl ethyl ketone	Fruity	0.85±0.04^{a}	0.58±0.03^{b}	0.62±0.03^{b}	-	0.80±0.06^{a}	0.68±0.04^{b}
	2, 3-butanedione	Butter, sweet, caramel	1.36±0.07^{c}	1.73±0.10^{b}	1.64±0.17^{b}	2.33±0.15^{a}	2.20±0.13^{a}	0.63±0.07^{d}
	2, 3-hexadione	Sweet, caramel, butter	1.78±0.14^{b}	1.85±0.15^{b}	1.97±0.12^{ab}	2.16±0.15^{a}	2.25±0.11^{a}	1.70±0.16^{b}
	3, 4-hexanedione	Butter, almond, nutty	1.28±0.09^{a}	0.67±0.05^{c}	0.85±0.09^{b}	0.83±0.07^{b}	0.93±0.09^{b}	0.81±0.04^{b}
	ethylcyclopentenone	Sweet, caramel, maple	2.01±0.10^{b}	2.05±0.14^{b}	2.06±0.15^{b}	1.99±0.20^{b}	2.16±0.14^{b}	2.59±0.13^{a}
	3-hexanone	Sweet, fruit, waxiness	0.54±0.03^{a}	-	0.25±0.04^{b}	-	-	-
	2, 3-pentadione	Butter, caramel, caramel, nutty	7.71±0.50^{b}	6.49±0.26^{d}	8.29±0.34^{ab}	8.47±0.41^{ab}	8.90±0.37^{a}	6.90±0.42^{c}
	(E) -beta-damascenone	Apple, rose, honey, sweet	-	-	-	-	-	0.45±0.04^{a}
	Furfural acetone	Spicy, cinnamon, vanilla	-	-	-	-	-	0.41±0.03^{a}
	Total		15.53±1.16^{b}	13.37±1.43^{c}	15.67±1.29^{b}	15.78±1.35^{b}	17.26±1.54^{a}	14.17±1.09^{bc}
Pyridine	pyridine	Sour, pourri	12.86 ±0.53^{b}	12.85 ±0.62^{b}	13.74 ±0.57^{ab}	14.12 ±0.65^{a}	14.70 ±0.49^{a}	12.57 ±0.62^{b}
	3-ethylpyridine	Tobacco, leather	-	0.43±0.04^{b}	0.37±0.04^{b}	0.57±0.05^{a}	-	-
	Total		12.86±1.06^{ab}	13.29±1.29^{ab}	14.11±0.98^{a}	14.70±1.15^{a}	14.70±1.43^{a}	12.57±1.11^{b}
Phenols	phenol	Plastics, caoutchouc	0.19±0.01^{f}	0.52±0.04^{e}	1.11±0.06^{d}	1.41±0.08^{c}	1.75±0.11^{b}	2.17±0.19^{a}
	4-ethyl guaiacol	Spicy, smoky, bacon	1.02±0.08^{c}	1.09±0.10^{bc}	1.25±0.13^{b}	1.16±0.11^{b}	1.18±0.10^{b}	1.98±0.15^{a}
	o-cresol	Musty, plastics, herbs	-	0.61±0.05^{b}	0.49±0.06^{b}	0.54±0.05^{b}	0.58±0.07^{b}	0.80±0.06^{a}
	Total		1.21±0.05^{e}	2.22±0.08^{d}	2.83±0.11^{c}	3.11±0.19^{bc}	3.51±0.23^{b}	4.96±0.30^{a}
Pyrrole	1-methylpyrrole	Smoky, woody, herbs	2.48±0.10^{a}	1.27±0.08^{c}	1.33±0.09	2.74±0.20^{a}	1.55±0.19^{b}	1.24±0.13^{c}
	2-acetyl-1-methylpyrrole	Earthy	2.68±0.12^{c}	3.34±0.19^{b}	3.19±0.20^{b}	2.76±0.18^{c}	3.22±0.16^{b}	4.06±0.29^{a}
	1-furfuryl pyrrole	Plastics, green, waxiness,	7.47±0.43^{bc}	7.37±0.36^{bc}	7.56±0.52^{bc}	7.01±0.43^{c}	8.00±0.50^{b}	9.33±0.55^{a}

		vegetables						
	2-acetylpyrrole	Musty, cherry	1.20±0.10^{a}	0.75±0.06^{c}	0.61±0.05^{d}	0.44±0.07^{e}	0.63±0.05^{d}	0.94±0.05^{b}
	2-formylpyrrole	Musty	0.85±0.05^{b}	0.91±0.07^{b}	0.69±0.05^{c}	0.43±0.04^{d}	0.82±0.07^{b}	1.24±0.10^{a}
	Total		14.68±1.05^{b}	13.66±1.23^{bc}	13.37±1.17^{c}	13.37±1.20^{c}	14.20±0.98^{bc}	16.82±1.03^{a}
Ethers	Furfuryl methyl ether	Roasted, coffee	2.44±0.15^{b}	2.26±0.18^{b}	2.34±0.19^{b}	2.82±0.16^{a}	2.56±0.20^{ab}	1.98±0.15^{c}
	Difurfuryl ether	Coffee, nutty, earthy	1.29±0.09^{c}	1.65±0.08^{b}	1.55±0.12^{bc}	1.44±0.10^{bc}	1.35±0.12^{c}	2.53±0.19^{a}
	Furfuryl methyl sulfide	Onion, garlic, sulphury	2.92±0.19^{a}	2.26±0.20^{b}	-	-	-	-
	Total		6.66±0.45^{a}	6.16±0.39^{a}	3.90±0.21^{c}	4.28±0.33^{bc}	3.91±0.29^{c}	4.54±0.34^{b}
	Total compounds		387.25±25.75^{b}	402.84±32.88^{ab}	410.23±27.43^{ab}	418.05±36.47^{ab}	449.40±39.66^{ab}	472.49±40.23^{a}

Values are expressed as mean ± standard deviation. The superscript letters (a, b, c, d, e, and f) represent statistically significant differences between extraction conditions, as determined through one-way analysis of variance ($p < 0.05$). Control group: conventional cold brew (0.1 MPa, 12 h, 5℃)

Table S2. Volatile components of UHP-assisted cold brew coffee at different times

Aroma Type	Compound Name	Aroma descriptor	Content/(μg/L)					
			Time/(min)					Control group
			10	15	20	25	30	
Furans	2-methylfuran	Chocolate	0.84±0.05^{d}	1.42±0.09^{b}	1.13±0.07^{c}	1.56±0.12^{b}	2.26±0.15^{a}	1.56±0.09^{b}
	2, 5-dimethylfuran	Meaty, beef, bacon	0.42±0.03^{c}	0.56±0.04^{b}	0.49±0.03^{bc}	0.28±0.02	0.66±0.04^{a}	0.3±0.02^{d}
	furfural	Sweet, almond, bread	77.83±5.64^{c}	78.47±5.42^{c}	80.53±5.74^{bc}	82.21±7.32^{bc}	94.99±5.65^{a}	86.46±4.85^{b}
	2-acetyl-5-methylfuran	Nutty, vanilla, coconut	-	0.54±0.04^{c}	0.48±0.04^{c}	0.77±0.05^{b}	-	0.98±0.09^{a}
	2-propionylfuran	Fruit	-	4.44±0.03^{b}	4.79±0.35^{ab}	4.31±0.42^{b}	-	5.06±0.35^{a}
	2-(furan-2-methyl-furan)	Roasted	2.32±0.15^{d}	2.65±0.21^{c}	2.58±0.17^{c}	2.76±0.12^{b}	3.08±0.26^{a}	3.16±0.24^{a}
	Furfuryl alcohol	Caramel	20.38±1.36^{c}	23.40±1.45^{bc}	23.51±1.93^{bc}	24.45±2.11^{b}	37.03±2.65^{a}	25.33±2.01^{b}
	2-acetylfuran	Sweet, almond, cocoa, caramel	15.55±0.86^{c}	17.28±0.95^{b}	17.44±1.38^{b}	17.71±1.19^{b}	19.90±1.58^{a}	19.91±1.25^{a}
	Total		117.34±9.88^{c}	128.76±8.46^{bc}	130.93±11.52^{bc}	134.04±10.24^{bc}	157.91±12.44^{a}	142.74±11.29^{ab}
Aldehydes	2-methylbutyral	Cocoa, coffee, nutty, malty, alcoholic	1.93±1.28^{d}	8.23±0.59^{bc}	7.59±0.51^{c}	9.10±0.88^{a}	8.37±0.57^{bc}	9.07±0.81^{a}
	isoamylaldehyde	Chocolate	-	4.38±0.25^{d}	6.81±0.34^{c}	7.74±0.41^{b}	8.63±0.68^{a}	7.8±0.64^{ab}
	Hexal (aldehyde C-6)	Green, fruity	0.63±0.05^{b}	0.73±0.06^{b}	1.11±0.07^{a}	1.04±0.06^{a}	0.64±0.05^{b}	0.39±0.02^{c}
	benzaldehyde	Bitter almond odor, nutty	2.69±0.15^{a}	2.02±0.13^{c}	1.95±0.12^{c}	2.32±0.18^{b}	2.30±0.16^{b}	2.53±0.25^{ab}
	5-methylfurfural	Spicy, caramel, maple	66.48±5.32^{b}	67.45±4.27^{b}	66.64±5.34^{b}	68.39±5.87^{b}	88.68±7.51^{a}	80.26±6.33^{ab}

	1-methyl-2-pyrroformaldehyde	Roasted, nutty	9.58±0.70^{b}	9.78±0.64^{b}	10.20±0.82^{b}	10.81±0.93^{b}	12.81±0.81^{a}	11.85±0.92^{ab}
	Total		81.32±5.35^{d}	92.59±6.87^{cd}	94.30±7.55^{cd}	99.40±8.45^{bc}	121.43±10.45^{a}	111.89±9.79^{ab}
Esters	Glycol diacetate	Green, flowery, alcoholic	1.76±0.11^{b}	1.63±0.13^{b}	1.61±0.10^{b}	2.68±0.19^{a}	-	2.60±0.19^{a}
	Furfuryl acetate	Sweet, fruit, banana	59.78±3.18^{c}	67.01±6.43^{b}	67.21±4.83^{b}	68.52±5.61^{b}	75.17±5.68^{ab}	78.61±6.99^{a}
	Furfuryl propionate	Sweet, fruit, banana	2.84±0.17^{c}	3.17±0.25^{b}	3.11±0.19^{b}	3.24±0.17^{b}	3.92±0.35^{a}	4.35±0.34^{a}
	Total		64.38±4.74^{c}	71.81±6.52^{bc}	71.94±5.95^{bc}	74.43±6.65^{abc}	79.09±5.41^{ab}	85.57±6.79^{a}
Pyrazines	2, 5-dimethylpyrazine	Cocoa, nutty, beef	6.08±0.59^{d}	7.11±0.56^{c}	7.82±0.64^{b}	8.39±0.71^{ab}	9.53±0.81^{a}	8.33±0.81^{ab}
	2, 6-dimethylpyrazine	Cocoa, nutty	6.09±0.41^{c}	7.20±0.39^{b}	7.19±0.61^{b}	8.00±0.55^{a}	8.41±0.59^{a}	7.89±0.42^{a}
	2-ethylpyrazine	Peanut, butter, nutty, cocoa	4.42±0.39^{c}	4.73±0.35^{c}	6.74±0.55^{b}	7.76±0.72^{a}	6.92±0.43^{b}	7.36±0.48^{ab}
	2, 3-dimethylpyrazine	Nutty, cocoa, peanut, walnut	0.54±0.04^{c}	0.86±0.06^{b}	0.99±0.06^{a}	0.58±0.03^{c}	1.00±0.08^{a}	0.61±0.05^{c}
	2-ethyl-6-methylpyrazine	Roasted, potato	10.53±0.56^{e}	11.44±0.84^{d}	12.96±0.75^{c}	15.00±0.76^{b}	16.15±0.97^{a}	15.88±0.91^{ab}
	Hazelnut pyrazine	Nutty, peanut, corn, bread	3.13±0.30^{d}	4.14±0.35^{c}	4.97±0.38^{b}	5.08±0.47^{b}	6.02±0.58^{a}	6.09±0.53^{a}
	3, 6-theopyrazine	Potato, cocoa, nutty	5.74±0.41^{c}	5.63±0.42^{c}	4.96±0.43^{d}	6.37±0.51^{ab}	5.94±0.30^{bc}	7.20±0.62^{a}
	2-vinylpyrazine	Nutty	-	0.58±0.04	-	-	-	-
	Coffee pyrazine	Coffee, nutty	1.24±0.07	-	-	-	-	-
	Total		37.77±2.75^{c}	41.69±3.43^{bc}	45.63±4.21^{b}	51.18±3.95^{a}	53.98±4.02^{a}	53.34±4.15^{a}
Alcohols	hexanol	Fruit, alcoholic, sweet, green	-	0.71±0.05^{b}	0.85±0.05^{a}	0.81±0.06^{a}	-	0.69±0.04^{b}

	(E). -linalool oxide (furanoic acid)	Flowery	4.55±0.34^{c}	5.35±0.35^{b}	5.78±0.48^{b}	5.73±0.42^{b}	8.33±0.81^{a}	7.75±0.61^{a}
	2-ethyl-1-hexanol	Oranges, flowery, sweet		0.60±0.03^{a}	0.59±0.02^{a}	0.65±0.04^{a}	-	0.59±0.04^{a}
	linalool	Oranges, flowery, sweet, rose	6.95±0.55^{c}	7.02±0.61^{c}	7.79±0.63^{c}	9.38±0.48^{b}	9.93±0.91^{b}	12.93±0.93^{a}
	alpha-terpineol	Pine, terpene, lilac, oranges, flowery	2.32±0.17^{c}	2.31±0.20^{c}	2.10±0.15^{d}	2.18±0.20^{d}	2.87±0.15^{b}	3.24±0.28^{a}
	nerolol	Sweet, oranges	0.51±0.05^{b}	0.52±0.03^{b}	0.39±0.05^{c}	0.40±0.02^{c}	0.68±0.04^{a}	0.69±0.07^{a}
	Total		14.33±1.21^{d}	16.51±1.43^{c}	17.52±1.36^{bc}	19.16±1.55^{b}	21.81±1.38^{ab}	25.89±2.02^{a}
Ketones	Methyl ethyl ketone	Fruit, camphor	0.25±0.02^{b}	0.28±0.02^{b}	0.62±0.04^{a}	0.26±0.02^{b}	-	0.68±0.05^{a}
	2, 3-butanedione	Butter, sweet, caramel	-	1.72±0.13^{a}	1.64±0.12^{a}	1.41±0.10^{b}	1.73±0.09^{a}	0.63±0.04^{c}
	2, 3-hexadione	Sweet, caramel, butter, fruit	1.15±0.01^{d}	2.75±0.16^{a}	1.97±0.18^{b}	1.64±0.13^{c}	1.88±0.14^{b}	1.70±0.12^{c}
	3, 4-hexanedione	Butter, almond, nutty, caramel	1.31±0.07^{b}	1.45±0.10^{b}	0.85±0.05^{c}	0.72±0.03^{d}	1.63±0.11^{a}	0.81±0.08^{cd}
	ethylcyclopentenone	Sweet, caramel, maple	1.98±0.12^{c}	2.13±0.19^{c}	2.06±0.13^{c}	1.97±0.16^{c}	2.93±0.28^{a}	2.59±0.21^{b}
	3-hexanone	Sweet, fruit, grape	-	0.23±0.01^{b}	0.25±0.02^{b}	1.13±0.08^{a}	-	-
	2, 3-pentadione	Butter, caramel, nutty	3.81±0.32^{d}	5.64±0.47^{c}	8.29±0.43^{a}	6.37±0.52^{bc}	6.11±0.54^{bc}	6.90±0.52^{b}
	1-acetoxyacetone	Fruit, butter, nutty	-	-	-	-	2.49±0.16	-
	(E) -beta-damascenone	Apple, rose, honey, sweet	-	-	-	-	-	0.45±0.03
	2, 3-octanedione	Cumin, coriander	0.68±0.04	-	-	-	-	-
	Furfural acetone	Spicy, cinnamon, vanilla	-	-	-	-	-	0.41±0.04
	Total		9.17±0.62^{c}	14.20±1.09^{b}	15.68±1.25^{ab}	13.50±1.16^{b}	16.77±1.34^{a}	14.17±1.29^{b}

Pyridines	pyridine	Sour, pourri	9.12±0.57^{c}	11.16±0.83^{b}	13.74±0.93^{a}	13.21±0.69^{a}	12.66±0.69^{ab}	12.57±0.75^{ab}
	3-ethylpyridine	Tobacco, leather	-	-	0.37±0.03^{a}	-	0.37±0.02^{a}	-
	Total		9.12±0.59^{d}	11.16±0.83^{c}	14.11±1.26^{a}	13.21±1.21^{ab}	13.03±1.09^{ab}	12.57±0.93^{b}
Phenols	phenol	Plastics, caoutchouc	0.74±0.06^{e}	0.85±0.05^{d}	1.11±0.07^{c}	1.22±0.09^{bc}	1.47±0.13^{b}	2.17±0.18^{a}
	4-ethyl guaiacol	Spicy, smoky, bacon	1.19±0.08^{b}	1.22±0.07^{b}	1.25±0.10^{b}	1.27±0.11^{b}	1.99±0.14^{a}	1.98±0.14^{a}
	o-cresol	Musty, plastics, herbs	0.53±0.03^{c}	0.62±0.04^{b}	0.49±0.04^{c}	0.50±0.03^{c}	0.81±0.05^{a}	0.80±0.05^{a}
	Total		2.47±0.17^{d}	2.69±0.15^{cd}	2.85±0.19^{c}	2.99±0.16^{c}	4.27±0.25^{b}	4.96±0.33^{a}
Pyrroles	1-methylpyrrole	Smoky, woody, herbs	1.97±0.14^{b}	1.47±0.11^{c}	1.33±0.11^{cd}	1.44±0.12^{c}	2.43±0.18^{a}	1.24±0.10^{d}
	2-acetyl-1-methylpyrrole	Earthy	3.04±0.26^{b}	3.05±0.28^{b}	3.19±0.25^{b}	3.34±0.19^{b}	4.15±0.35^{a}	4.06±0.38^{a}
	1-furfuryl pyrrole	Plastics, waxiness, fruit, vegetables	7.04±0.45^{c}	7.58±0.64^{bc}	7.56±0.65^{bc}	7.90±0.51^{b}	9.86±0.66^{a}	9.33±0.69^{a}
	2-acetylpyrrole	Musty, nutty	0.57±0.04^{b}	0.60±0.05^{b}	0.61±0.04^{b}	0.60±0.03^{b}	1.59±0.13^{a}	0.94±0.08^{b}
	2-formylpyrrole	Musty	0.66±0.05^{cd}	0.62±0.04^{d}	0.69±0.06^{cd}	0.73±0.04^{c}	1.85±0.11^{a}	1.24±0.12^{b}
	Total		13.27±1.34^{c}	13.31±0.98^{c}	13.37±1.25^{c}	14.00±1.21^{c}	19.89±1.43^{a}	16.82±1.39^{b}
Ethers	Furan, 2-(methoxymethyl)-	Coffee	-	-	2.34±0.21^{a}	2.32±0.18^{a}	2.04±0.12^{ab}	1.98±0.18^{b}
	Furan, 2,2'-[oxybis(methylene)]bis-	Coffee, nutty, earthy	1.59±1.21^{b}	1.69±0.13^{b}	1.55±0.14^{b}	1.70±0.09^{b}	2.61±0.22^{a}	2.53±0.24^{a}
	Furan, 2-[(methylthio)methyl]-	Onion, garlic, sulphury, vegetables	-	1.77±0.11	-	-	-	-
	Total		1.59±0.12^{d}	3.46±0.25^{c}	3.90±0.33^{b}	4.02±0.38^{ab}	4.65±0.42^{a}	4.54±0.39^{a}
Total compounds			350.77±28.92^{d}	396.17±33.45^{cd}	410.23±38.42^{bcd}	438.07±36.99^{abc}	507.84±43.68^{a}	472.49±40.01^{ab}

Values are expressed as mean ± standard deviation. The superscript letters (a, b, c, d, and e) represent statistically significant differences between extraction conditions, as determined through one-way analysis of variance ($p < 0.05$). Control group: conventional cold brew (0.1 MPa, 12 h, 5℃)
